# Hybrid volumetric modulated arc therapy for hypofractionated radiotherapy of breast cancer: a treatment planning study

**DOI:** 10.1007/s00066-020-01696-8

**Published:** 2020-10-17

**Authors:** Alexander Venjakob, Michael Oertel, Dominik Alexander Hering, Christos Moustakis, Uwe Haverkamp, Hans Theodor Eich

**Affiliations:** Department of Radiation Oncology, University Hospital Muenster, Building A1, 1 Albert-Schweitzer-Campus, 48149 Muenster, Germany

**Keywords:** Hybrid planning, Breast radiation therapy, Treatment planning challenge, Hypofractionation, Clinical application

## Abstract

**Purpose:**

This study aims to evaluate the best possible practice using hybrid volumetric modulated arc therapy (H-VMAT) for hypofractionated radiation therapy of breast cancer. Different combinations of H‑VMAT—a combination of three-dimensional radiotherapy (3D-CRT) and VMAT—were analyzed regarding planning target volume (PTV), dose coverage, and exposure to organs at risk (OAR).

**Methods:**

Planning computed tomography scans were acquired in deep-inspiration breath-hold. A total of 520 treatment plans were calculated and evaluated for 40 patients, comprising six different H‑VMAT plans and a 3D-CRT plan as reference. H‑VMAT plans consisted of two treatment plans including 3D-CRT and VMAT. During H‑VMAT planning, the use of hard wedge filters (HWF) and beam energies were varied. The reference plans were planned with mixed beam energies and the inclusion/omission of HWF.

**Results:**

Compared to the reference treatment plans, all H‑VMAT plans showed consistently better PTV dose coverage, conformity, and homogeneity. Additionally, OAR protection was significantly improved with several H‑VMAT combinations (*p* < 0.05). The comparison of different H‑VMAT combinations showed that inclusion of HWF in the base plan had a negative impact on PTV dose coverage, conformity, and OAR exposure. It also increased the planned monitor units and beam-on time. Advantages of using lower beam energies (6-MV photons) in both the base plan and in the VMAT supplementary dose were observed.

**Conclusion:**

The H‑VMAT technique is an effective possibility for generating homogenous and conformal dose distributions. With the right choice of H‑VMAT combination, superior OAR protection is achieved compared to 3D-CRT.

## Introduction

Radiotherapy (RT) is pivotal in the adjuvant treatment of breast cancer, improving both local control and overall survival [1, 2]. Various radiation concepts and techniques have been established over time. Static three-dimensional radiotherapy (3D-CRT) represents the conventional radiation technique, whereby tangential opposing fields with hard wedge filters (HWF) are used. Modern dynamic irradiation techniques, such as intensity-modulated radiation therapy (IMRT) and volumetric modulated arc therapy (VMAT), attempt to generate more homogeneous and conformal dose distributions for the planning target volume (PTV). Furthermore, better protection of organs at risk (OAR) may be achieved [3, 4]. Nevertheless, dynamic radiation techniques bear the risk of increased induction of secondary tumors attributed to larger areas of low-dose exposure and increased monitor units (MU) [5]. To balance the respective advantages of static and dynamic radiation techniques, Mayo et al. [6] have developed a composite approach combining 3D-CRT and IMRT named hybrid intensity-modulated radiation therapy (H‑IMRT). In our study, we examined the impact of different variations with respect to HWF in the 3D-CRT base plan and beam energies for the application of hybrid volumetric arc therapy (H‑VMAT). Patients were evaluated in terms of PTV dose coverage and OAR exposure. Concerning OAR, dose comparisons were established and differences between left- and right-sided breast therapy were analyzed.

## Materials and methods

### Patient selection, positioning, and computed tomography

The patients included in this study were selected by defining a volume-based standardized breast size (*n* = 200), whereby the PTV sizes were evaluated as a measure of breast size. The computed tomography (CT) scans (Canon Aquilion LB, Canon Medical Systems Europe B.V., Zoetermeer, the Netherlands) were acquired with a slice thickness of 3.0 mm. Patients were positioned in a headfirst supine position. An arm board helped to immobilize the chest and thorax with the arms positioned overhead. To minimize intrafractional movement, the CT scans were carried out in deep-inspiration breath-hold (DIBH). This method bears dosimetric advantages, especially for cardiopulmonary OAR exposure and secondary lung cancer risk [7–10]. Of the 200 patients analyzed, 110 patients met the inclusion criteria of the study regarding PTV size and no exclusion criteria were included, such as funnel chests, pathological enlarged hearts, or anatomic variations. Out of these patients, 40 (20 per breast side) were selected randomly.

### Treatment planning and techniques

The Eclipse software (version 15.6, Varian Medical Systems, Palo Alto, CA, USA) was used for treatment planning. Treatment plans were created for a Varian TrueBeam linear accelerator, the calculation model was based on the anisotropic analytical algorithm (AAA, version 15.5.12) and a calculation grid size of 2.5 mm. Each PTV included the whole breast and was cropped 5 mm from the skin surface, resulting in a VMAT optimization where the dose is not forcibly modulated to the surface. The thoracic wall was defined as part of the PTV. Individual treatment plans were created using the H‑VMAT technique and the 3D-CRT technique as reference (Ref). The H‑VMAT plans represented sum plans: each consisted of two treatment plans including 3D-CRT and VMAT. The prescribed dose was 40.05 Gy in 15 fractions (2.67 Gy per fraction) based on the START B trail, which demonstrated the non-inferiority of this hypofractionated regime regarding locoregional control and toxicity in comparison to normofractionated treatment [11]. The reference treatment plans were planned as tangential opposing fields with mixed beam energies (6 MV and 15 MV) and HWF. All planning parameters such as gantry and wedge filter angles, collimator position, and field weightings were individually optimized. H‑VMAT plans were weighted 80/20 between 3D-CRT and VMAT, which included 3D-CRT as a base plan for VMAT optimization. The supplementary dose consisted of a single VMAT field with an identical isocenter position. Its range of rotation angle corresponded to the tangential angles from the base plan. The collimator rotation angle was set to 5° and the maximum dose rate to 600 MU/min. A standard optimization protocol was used for the inverse planning process of VMAT, which was adapted individually. Flattening-filter beams were used in all treatment plans. Within the hybrid approach, six different H‑VMAT combinations (HV1–HV6, Table 1) were used, varying beam energy and the application or omission of HWF in the 3D-CRT plans. The selection of these combinations was based on a previous evaluation by our group. This previous study was an initial assessment of 32 different combinations, 16 of which were within the weightings 60/40 and 80/20, which were evaluated based on a case number of 5 patients. To assess the optimal hybrid combination, superior combinations of this evaluation with respect to quality indices (PTV coverage, conformity, homogeneity) and OAR doses were analyzed in detail (Table 1).

**Table 1:** Definition of irradiation techniques using different H‑VMAT constellations and conventional 3D-CRT as a reference treatment plan

IT/H-VMAT constellation	3D-CRT base plan energy (MV)	Individual hard wedge filters (base plan)	VMAT energy (MV)
Ref	6/15 (mixed on both sides)	Yes	–
HV1	6/15 (mixed on both sides)	No	6
HV2	6	No	6
HV3	6	No	15
HV4	6	Yes	6
HV5	6	Yes	15
HV6	6 (posterior lateral), 15 (anterior medial)	No	6

*IT* irradiation techniques, *H‑VMAT *hybrid volumetric modulated arc therapy, *HV1–HV6 *H‑VMAT constellations, *3D-CRT *conventional three-dimensional radiotherapy, *Ref* reference treatment plan

PTV dose coverage had the first priority in treatment planning. The PTV dose requirements in our study were based on the recommendations of ICRU Report 83 [12]:

$$\mathrm{PTV} \text{D}_{\text{mean}}=40.05\,\mathrm{Gy}(100 \% )$$

$$38.05\,\mathrm{Gy}(95\mathrm{\% })\leq \text{PTV D}_{95\mathrm{\%}}\leq 38.85\,\mathrm{Gy}(97\mathrm{\% })$$

$$\mathrm{PTV} \text{D}_{2\mathrm{\% }}\leq 42.85\,\mathrm{Gy}(107\mathrm{\% })$$

In order to ensure an appropriate plan comparison with regard to OAR doses, these PTV requirements should be kept as consistent as possible. For this purpose, an upper limit for PTV D_95%_ was set. Minimizing OAR doses was a secondary priority. The protection was implemented so that the PTV dose requirements could be guaranteed. Each of the prepared 520 treatment plans was separately optimized for individual patients.

### Dosimetric parameters

Four different indices were used to assess PTV dose conformity and homogeneity:

#### 1. Coverage index [13]

$$\mathrm{COV}=\frac{\mathrm{PTV}_{\mathrm{PI}}}{\mathrm{PTV}}(\text{ideal value}\,1.000)$$

#### 2. External index [14]

$$\mathrm{EI}=\frac{\mathrm{TV}_{\mathrm{PI}}-\mathrm{PTV}_{\mathrm{PI}}}{\mathrm{PTV}}(\text{ideal value}\,0.000)$$

#### 3. Conformation number [15]

$$\text{CN}=\frac{\mathrm{PTV}_{\mathrm{PI}}}{\mathrm{PTV}}\mathrm{*}\frac{\mathrm{PTV}_{\mathrm{PI}}}{\mathrm{TV}_{\mathrm{PI}}}(\text{ideal value}\,1.000)$$

#### 4. Homogeneity index [12]

$$\mathrm{HI}=\frac{\text{D}_{2}-\text{D}_{98}}{\text{D}_{\text{mean}}}(\text{ideal value}\ 0.000)$$

PTV =: planning target volume (cm^3^)
PTV_PI_ =: partial volume in the PTV with at least the prescribed dose (cm^3^)
TV_PI_ =: partial volume in the whole body with at least the prescribed dose (cm^3^)
D_2_ =: near maximum dose: dose in 2% of PTV (Gy)
D_98_ =: near minimum dose: dose in 98% of PTV (Gy)
D_mean_ =: mean dose of the PTV (Gy) [16].

The Radiation Therapy Oncology Group (RTOG) Report 1005 [17] served as a guide for dosimetric evaluations of the OAR doses (for detailed OAR constraints, see Table 2). Our previous evaluation showed clinically negligible doses to the contralateral lung and spinal cord, so that these OAR were not considered in this study.

**Table 2:** Dose constraints of OAR defined in RTOG Report 1005 [17] for hypofractionated breast radiation therapy

OAR	Dose specifications (per protocol, variation acceptable)
Ipsilateral lung	V16 = 15–20%
	V8 = 35–40%
	V4 = 50–55%
Heart	D_mean_ = 320–400 cGy
Contralateral breast	D_5_ = 144–240 cGy

*OAR* organ at risk, *RTOG* Radiation Therapy Oncology Group, *VX* volume (%) of the OAR exposed to at least X Gy, *D*_5_ minimal dose (Gy) in 5% of the OAR volume with the highest exposure, *D*_mean_ mean dose

Further evaluation criteria were the planned monitor units (MU) and beam-on time (BOT) that were observed over the total number of patients.

SPSS software (for Windows, version 26, IBM, Armonk, NY, USA) was used for the statistical analysis. A two-sided Wilcoxon rank-sum test for paired samples was used to evaluate dosimetric parameters. A *p*-value <0.05 was considered statistically significant.

## Results

### Planning target volume sizes

A volume-specific standard breast of 961 ± 346 cm^3^ was determined from the data of 200 patients. A Kolmogorov–Smirnov test confirmed the presence of a normal sample distribution. The analysis included only patients with a PTV or breast size within the range of the standard deviation, integrating 68% of all women, whereas anatomical variations such as funnel chests and pathological enlarged hearts were not considered.

### Planning target volume dose coverage

**Fig. 1:**
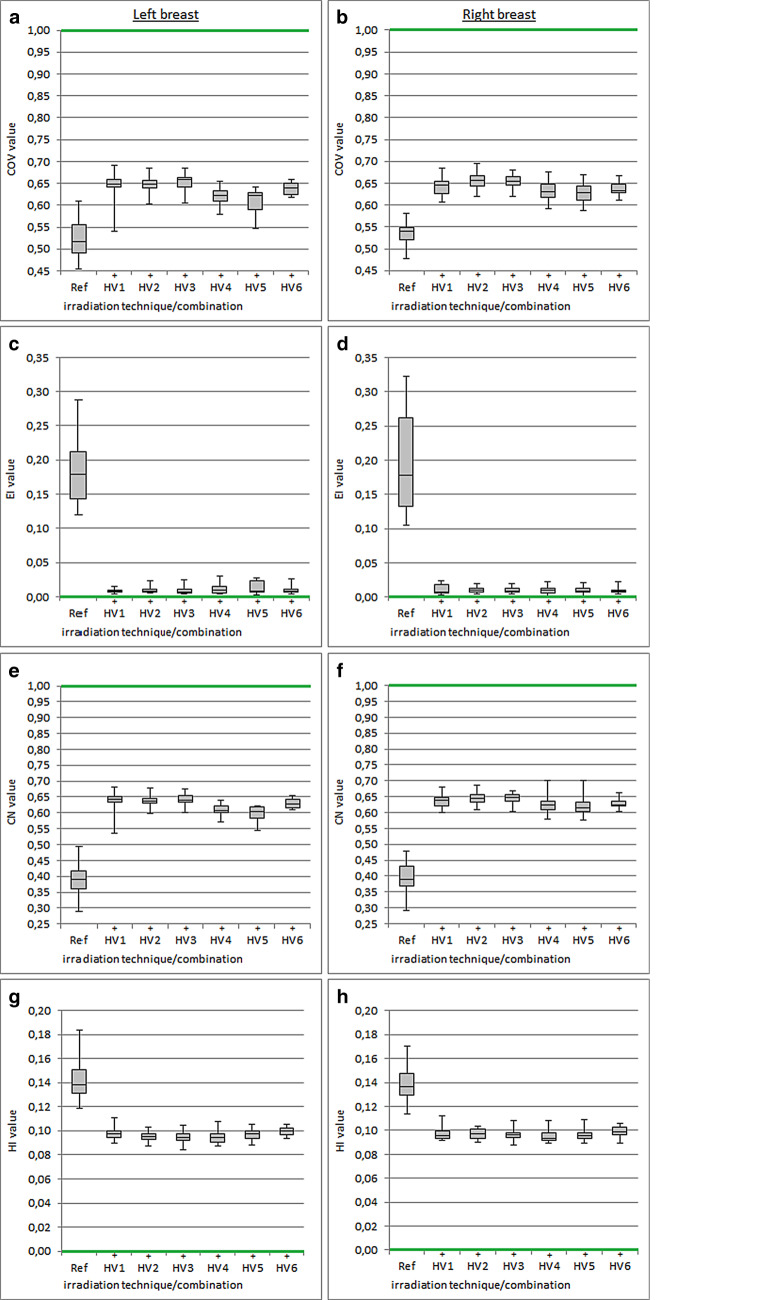
Boxplots of indexes evaluating planning target volume dose coverage (*COV*: **a**, **b**), exposure of normal tissue (*EI*: **c**, **d**), conformity (*CN*: **e**, **f**), and homogeneity (*HI*: **g**, **h**) using different hybrid volumetric modulated arc therapy (H-VMAT) constellations (*HV1–HV6*) and conventional three-dimensional radiotherapy as a reference treatment plan (Ref). Significant differences of the respective H‑VMAT combination compared to Ref are indicated by (+, advantage). The colored markings (*green*) correspond to the ideal values of the indices

Compared to the reference treatment plan, all analyzed H‑VMAT combinations consistently delivered significantly better values for PTV dose coverage (Fig. 1, Table 3). With the conventional radiation technique (Ref), dose coverage was consistently less successful than with H‑VMAT. Compared to Ref, all H‑VMAT combinations achieved significantly better EI values. The use of HWF in H‑VMAT was disadvantageous for the EI in left-sided breast therapy, whereby HV4 (EI = 0.012 ± 0.008) and HV5 (EI = 0.013 ± 0.009) showed worse values compared to other combinations without HWF use such as HV1 (EI = 0.008 ± 0.003). Compared to Ref (left CN = 0.391 ± 0.050, right CN = 0.395 ± 0.046), the evaluation of conformity showed better values in all H‑VMAT combinations. HV4 (left CN = 0.611 ± 0.016, right CN = 0.622 ± 0.021) and HV5 (left CN = 0.598 ± 0.025, right CN = 0.617 ± 0.020) revealed inferior results compared to other H‑VMAT combinations for both breast sides. Better dose homogeneity within the PTV (HI) was generally achieved in the H‑VMAT plans.

**Table 3:** Representation of the considered dosimetric parameters using H‑VMAT combinations and the reference treatment plan

Breast side	Dosimetric parameter	Radiation technique/H-VMAT combination^a^						
		Ref	HV1	HV2	HV3	HV4	HV5	HV6
Left	COV	0.523 ± 0.042	0.645 ± 0.029 (*p* < 0.0001)	0.647 ± 0.017 (*p* < 0.0001)	0.653 ± 0.019 (*p* < 0.0001)	0.622 ± 0.018 (*p* < 0.0001)	0.610 ± 0.028 (*p* < 0.0001)	0.639 ± 0.014 (*p* < 0.0001)
	EI	0.181 ± 0.041	0.008 ± 0.003 (*p* < 0.0001)	0.011 ± 0.006 (*p* < 0.0001)	0.010 ± 0.007 (*p* < 0.0001)	0.012 ± 0.008 (*p* < 0.0001)	0.013 ± 0.009 (*p* < 0.0001)	0.009 ± 0.005 (*p* < 0.0001)
	CN	0.391 ± 0.050	0.637 ± 0.029 (*p* < 0.0001)	0.637 ± 0.018 (*p* < 0.0001)	0.642 ± 0.020 (*p* < 0.0001)	0.611 ± 0.016 (*p* < 0.0001)	0.598 ± 0.025 (*p* < 0.0001)	0.630 ± 0.015 (*p* < 0.0001)
	HI	0.143 ± 0.018	0.098 ± 0.005 (*p* < 0.0001)	0.095 ± 0.004 (*p* < 0.0001)	0.095 ± 0.004 (*p* < 0.0001)	0.095 ± 0.005 (*p* < 0.0001)	0.097 ± 0.005 (*p* < 0.00001)	0.100 ± 0.004 (*p* < 0.00001)
	IL V16 (%)	14.9 ± 5.2	14.3 ± 5.0 (*p* < 0.0001)	13.9 ± 4.9 (*p* < 0.0001)	14.0 ± 4.9 (*p* < 0.0001)	14.2 ± 4.9 (*p* < 0.0001)	14.3 ± 4.9 (*p* < 0.0001)	14.4 ± 4.9 (*p* = 0.0005)
	IL V8 (%)	22.7 ± 6.4	23.1 ± 6.8 (*p* = 0.5437)	21.7 ± 6.5 (*p* = 0.0249)	21.9 ± 6.6 (*p* = 0.0971)	23.0 ± 6.1 (*p* = 0.5913)	23.3 ± 6.5 (*p* = 0.2942)	22.9 ± 6.8 (*p* = 0.7953)
	IL V4 (%)	44.4 ± 9.4	43.6 ± 10.8 (*p* = 0.5530)	41.1 ± 9.9 (*p* = 0.0028)	42.0 ± 10.3 (*p* = 0.0348)	46.4 ± 9.4 (*p* = 0.0259)	47.3 ± 10.4 (*p* = 0.0084)	43.0 ± 10.7 (*p* = 0.2730)
	IL D_mean_ (Gy)	8.5 ± 2.0	7.8 ± 1.9 (*p* < 0.0001)	7.4 ± 1.8 (*p* < 0.0001)	7.5 ± 1.8 (*p* < 0.0001)	8.1 ± 1.6 (*p* = 0.0023)	8.2 ± 1.7 (*p* = 0.0181)	7.7 ± 1.9 (*p* < 0.0001)
	Heart D_mean_ (cGy)	216 ± 42	128 ± 37 (*p* < 0.0001)	121 ± 34 (*p* < 0.0001)	123 ± 35 (*p* < 0.0001)	204 ± 33 (*p* = 0.0003)	205 ± 33 (*p* = 0.0005)	137 ± 39 (*p* < 0.0001)
	CB D_5_ (cGy)	200 ± 33	88 ± 26 (*p* < 0.0001)	70 ± 23 (*p* < 0.0001)	84 ± 29 (*p* < 0.0001)	176 ± 29 (*p* < 0.0001)	183 ± 32 (*p* < 0.0001)	90 ± 36 (*p* < 0.0001)
Right	COV	0.535 ± 0.028	0.644 ± 0.018 (*p* < 0.0001)	0.655 ± 0.019 (*p* < 0.0001)	0.655 ± 0.016 (*p* < 0.0001)	0.632 ± 0.022 (*p* < 0.0001)	0.627 ± 0.021 (*p* < 0.0001)	0.637 ± 0.015 (*p* < 0.0001)
	EI	0.197 ± 0.072	0.008 ± 0.003 (*p* < 0.0001)	0.010 ± 0.004 (*p* < 0.0001)	0.010 ± 0.004 (*p* < 0.0001)	0.010 ± 0.005 (*p* < 0.0001)	0.010 ± 0.005 (*p* < 0.0001)	0.009 ± 0.004 (*p* < 0.0001)
	CN	0.395 ± 0.046	0.636 ± 0.018 (*p* < 0.0001)	0.644 ± 0.019 (*p* < 0.0001)	0.645 ± 0.016 (*p* < 0.0001)	0.622 ± 0.021 (*p* < 0.0001)	0.617 ± 0.020 (*p* < 0.0001)	0.628 ± 0.015 (*p* < 0.0001)
	HI	0.140 ± 0.014	0.097 ± 0.006 (*p* < 0.0001)	0.097 ± 0.004 (*p* < 0.0001)	0.096 ± 0.005 (*p* < 0.0001)	0.096 ± 0.006 (*p* < 0.0001)	0.097 ± 0.005 (*p* < 0.0001)	0.099 ± 0.004 (*p* < 0.0001)
	IL V16 (%)	15.3 ± 3.7	15.3 ± 3.6 (*p* = 0.7427)	14.7 ± 3.6 (*p* < 0.0001)	14.8 ± 3.7 (*p* = 0.0001)	15.0 ± 3.6 (*p* = 0.0166)	15.0 ± 3.7 (*p* = 0.0129)	15.1 ± 3.6 (*p* = 0.1621)
	IL V8 (%)	23.4 ± 4.1	26.1 ± 4.8 (*p* = 0.0033)	23.2 ± 4.6 (*p* = 0.7689)	23.6 ± 4.7 (*p* = 0.6489)	24.6 ± 4.0 (*p* = 0.0240)	24.9 ± 4.9 (*p* = 0.0191)	25.1 ± 4.5 (*p* = 0.0178)
	IL V4 (%)	44.8 ± 5.6	49.0 ± 7.9 (*p* = 0.0107)	43.3 ± 7.8 (*p* = 0.2904)	44.4 ± 7.8 (*p* = 0.8226)	48.7 ± 7.8 (*p* = 0.0164)	49.3 ± 8.7 (*p* = 0.0180)	46.9 ± 7.4 (*p* = 0.1834)
	IL D_mean_ (Gy)	8.1 ± 1.2	8.2 ± 1.2 (*p* = 0.7931)	7.5 ± 1.2 (*p* = 0.0006)	7.6 ± 1.3 (*p* = 0.0059)	8.1 ± 1.1 (*p* = 0.8219)	8.1 ± 1.2 (*p* = 0.8098)	8.0 ± 1.1 (*p* = 0.4405)
	Heart D_mean_ (cGy)	132 ± 19	71 ± 26 (*p* < 0.0001)	63 ± 17 (*p* < 0.0001)	65 ± 20 (*p* < 0.0001)	133 ± 16 (*p* = 0.6923)	132 ± 20 (*p* = 0.8997)	78 ± 27 (*p* < 0.0001)
	CB D_5_ (cGy)	233 ± 45	116 ± 42 (*p* < 0.0001)	96 ± 35 (*p* < 0.0001)	111 ± 40 (*p* < 0.0001)	205 ± 38 (*p* < 0.0001)	213 ± 39 (*p* < 0.0001)	121 ± 51 (*p* < 0.0001)
Planned monitor units (MU)		490 ± 43	342 ± 27	365 ± 19	351 ± 40	540 ± 40	530 ± 40	342 ± 17
Planned beam-on time (min)		0.82 ± 0.07	0.91 ± 0.03	0.95 ± 0.03	0.94 ± 0.03	1.26 ± 0.06	1.25 ± 0.06	0.91 ± 0.03

*H‑VMAT *hybrid volumetric modulated arc therapy, *HV1–HV6 *H‑VMAT constellations, *Ref* reference treatment plan, *COV* planning target volume dose coverage, *EI* external index, *CN* conformation number, *HI* homogeneity index,* IL* ipsilateral, *CB* contralateral, *VX* volume (%) of the organ at risk (OAR) exposed to at least X Gy, *D*_5_ minimal dose (Gy) in 5% of the OAR volume with the highest exposure, *D*_mean_ mean dose

^a^HV1–HV6 are presented with *p*-values to demonstrate significant differences to Ref, using a two-sided Wilcoxon rank sum test for paired samples for the statistical analysis

The analysis within the hybrid combinations showed potential for improvement with regard to the quality indices (Table 4). The inclusion of HWF in the base plan (HV2/HV4 and HV3/HV5) resulted in significant disadvantages regarding COV and CN. The omission of HWF resulted in a significantly better homogeneity index in left-sided, but not in right-sided breast therapy (HV3/HV5). By using higher beam energies in the VMAT supplementary dose (HV2/HV3 and HV4/HV5), better CN values could be achieved. In addition, advantages in PTV dose coverage and conformity only in left-sided therapy (HV4/HV5) were observed.

**Table 4:** Representation of the considered dosimetric parameters using H‑VMAT combinations

Breast side	Dosimetric parameter	Supplementary dose energy 6 MV/15 MV^a^		Base plan without using HWF/using HWF^a^	
		HV2/HV3	HV4/HV5	HV2/HV4	HV3/HV5
Left	COV	0.1675	0.0055	<0.0001	<0.0001
	EI	0.8252	0.6079	0.3626	0.1987
	CN	0.1686	0.0038	<0.0001	<0.0001
	HI	0.4521	0.0319	0.7829	0.0196
	IL V16	0.1274	0.1139	<0.0001	<0.0001
	IL V8	0.3137	0.1323	0.0001	0.0005
	IL V4	0.1085	0.0356	<0.0001	<0.0001
	IL D_mean_	0.0696	0.0436	<0.0001	<0.0001
	Heart D_mean_	0.4134	0.4445	<0.0001	<0.0001
	CB D_5_	<0.0001	<0.0001	<0.0001	<0.0001
Right	COV	0.9909	0.0556	0.0005	<0.0001
	EI	0.5597	0.5217	0.8532	0.7558
	CN	0.9339	0.0491	0.0005	<0.0001
	HI	0.5291	0.4407	0.4650	0.8714
	IL V16	0.1995	0.5058	<0.0001	<0.0001
	IL V8	0.0646	0.5284	0.0007	0.0003
	IL V4	0.0413	0.5445	<0.0001	<0.0001
	IL D_mean_	0.0006	0.9428	<0.0001	0.0001
	Heart D_mean_	0.1249	0.7775	<0.0001	<0.0001
	CB D_5_	0.0001	0.0011	<0.0001	<0.0001

*H‑VMAT *hybrid volumetric modulated arc therapy, *HV5–HV5 *H‑VMAT constellations, *COV* planning target volume dose coverage, *EI* external index, *CN* conformation number, *HI* homogeneity index,* IL* ipsilateral, *CB* contralateral, *VX* volume (%) of the organ at risk (OAR) exposed to at least X Gy, *D*_5_ minimal dose (Gy) in 5% of the OAR volume with the highest exposure, *D*_mean_ mean dose

^*a*^*P*-values demonstrate the significant differences between the various H‑VMAT combinations, using a two-sided Wilcoxon rank sum test for paired samples for the statistical analysis. Values of the dosimetric parameters are listed in Table 3. HV2/HV3 and HV4/HV5 contain the same base plan, the supplementary dose energy was varied. HV2/HV4 and HV3/HV5 contain the same supplementary dose energy, the base plan was varied using HWF

### Organs at risk dose exposure

**Fig. 2:**
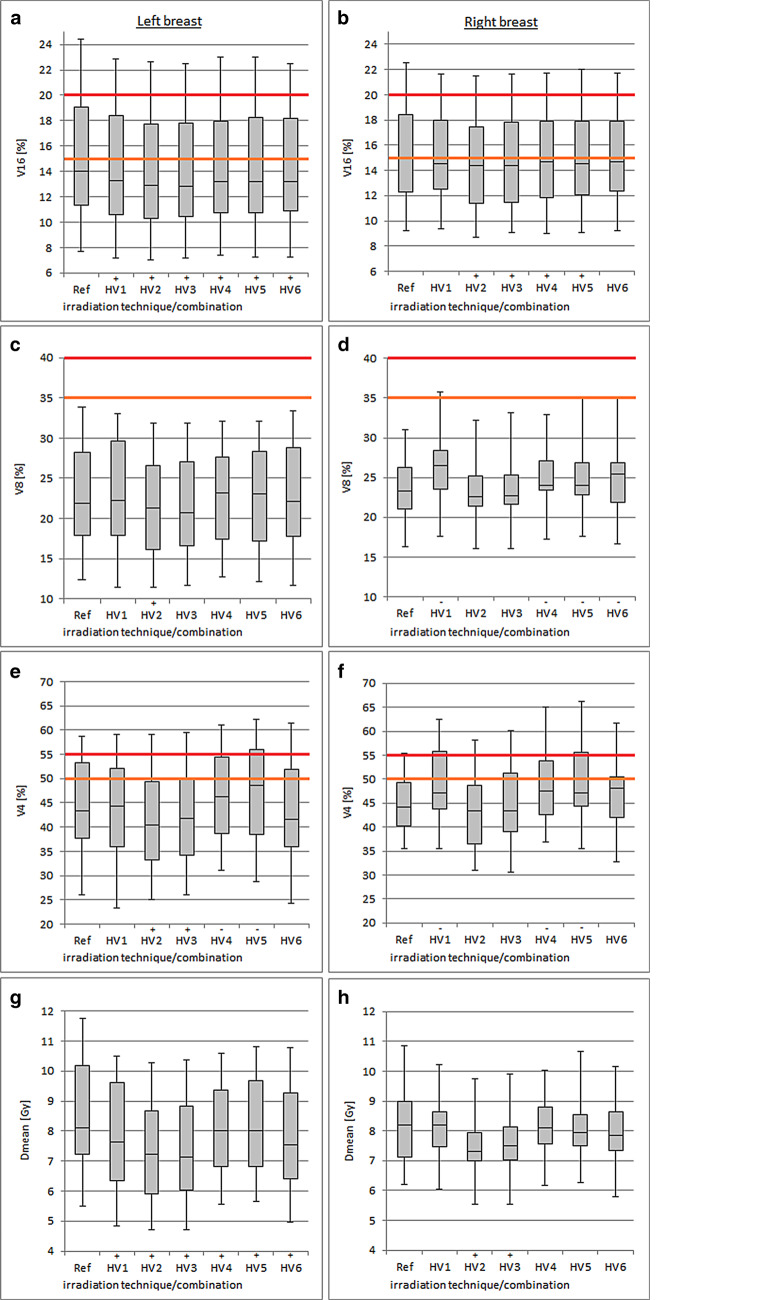
Boxplots of ipsilateral lung doses V16 (**a**, **b**), V8 (**c**, **d**), V4 (**e**, **f**), and D_mean_ (**g**, **h**) using different hybrid volumetric modulated arc therapy (H-VMAT) combinations (*HV1–HV6*) and conventional three-dimensional radiotherapy as the reference treatment plan (*Ref*). *VX* denotes the volume (%) of the organ at risk exposed with X Gy. The colored markings (*orange*: per protocol, *red*: variation acceptable) correspond to the dose constraints defined in Radiation Therapy Oncology Group Report 1005 [17] for hypofractionated breast radiation therapy. Significant differences of the respective H‑VMAT combination compared to Ref are indicated by (+, advantage) and (−, disadvantage)

Evaluation of the ipsilateral lung (IL) dose value V16 displayed significantly better values in almost all H‑VMAT combinations on both breast sides (Fig. 2, Table 3). Only for HV1 (V16 = 15.3 ± 3.6%) and HV6 (V16 = 15.1 ± 3.6%) in the case of right-sided breast irradiation was there no significant difference to the reference treatment plan (Ref V16 = 15.3 ± 3.7%). The IL V8 doses in left-sided breast irradiation were equivalent across all RT combinations, with only HV2 (V8 = 21.7 ± 6.5%) showing statistical benefit over Ref (V8 = 22.7 ± 6.4%). In right-sided breast irradiation, most hybrid combinations, HV1, HV4, HV5, and HV6, provided disadvantages. Overall, the tolerable dose values of the RTOG Report 1005 (V8 = 35–40%) were met in most used techniques (except for one patient HV1, HV5, and HV6 for the right breast). When considering the IL dose V4, only HV2 (V4 = 41.1 ± 9.9%) and HV3 (V4 = 42.0 ± 10.3%) were superior to Ref (V4 = 44.4 ± 9.4%) in left-sided therapy. HV4 (V4 = 46.4 ± 9.4%) and HV5 (V4 = 47.3 ± 10.4%) delivered significantly worse values as well as HV1, HV4, and HV5 on right-sided therapy. All other H‑VMAT combinations were equivalent to Ref regarding IL V4. The analysis of the mean ipsilateral lung dose showed advantages of the hybrid technique on left breast therapy, with significantly better values for H‑VMAT. In right-sided therapy, only H‑VMAT combinations HV2 (D_mean_ = 7.5 ± 1.2 Gy) and HV3 (D_mean_ = 7.6 ± 1.3 Gy) offered statistically significant advantages over the reference (D_mean_ = 8.1 ± 1.2 Gy).

**Fig. 3:**
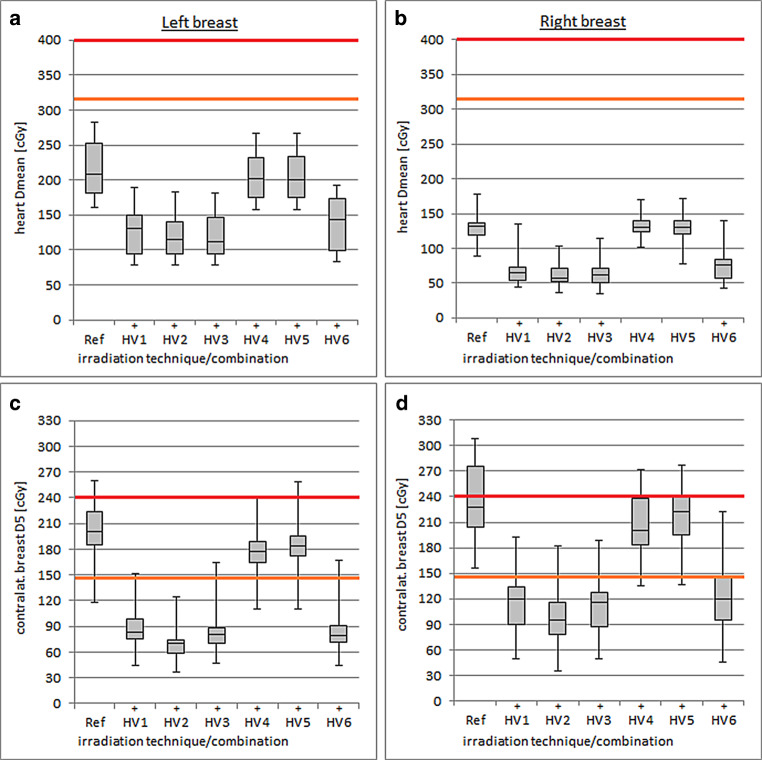
Boxplots of mean heart doses (**a**, **b**) and contralateral breast D_5_ doses (**c**, **d**) using different hybrid volumetric modulated arc therapy (H-VMAT) combinations (*HV1–HV6*) and conventional three-dimensional radiotherapy as the reference treatment plan (*Ref*). *DY* denotes the dose (Gy) of the organ at risk of Y%. The colored markings (*orange*: per protocol, *red*: variation acceptable) correspond to the dose constraints defined in Radiation Therapy Oncology Group Report 1005 [17] for hypofractionated breast radiation therapy. Significant differences of the respective H‑VMAT combination compared to Ref are indicated by (+, advantage)

All dose values of the heart complied with the dose specification of RTOG Report 1005 regarding D_mean_ = 320–400 cGy (Fig. 3, Table 3). Especially in left-sided breast therapy, the heart was better protected with all H‑VMAT combinations considered. HV1 (D_mean_ = 128 ± 37 cGy), HV2 (D_mean_ = 121 ± 34 cGy), HV3 (D_mean_ = 123 ± 35 cGy), and HV6 (D_mean_ = 137 ± 39 cGy) showed the best values and thus the most pronounced advantages over Ref (D_mean_ = 216 ± 42 cGy). In right-sided breast irradiation, the same trend could be observed (HV1 D_mean_ = 71 ± 26 cGy, HV2 D_mean_ = 63 ± 17 cGy, HV3 D_mean_ = 65 ± 20 cGy, HV6 D_mean_ = 78 ± 27 cGy, and Ref D_mean_ = 132 ± 19 cGy). The combinations HV4 and HV5 consistently achieved the highest heart exposure in H‑VMAT. In general, higher heart dose values were evaluated in left-sided breast therapy.

**Fig. 4:**
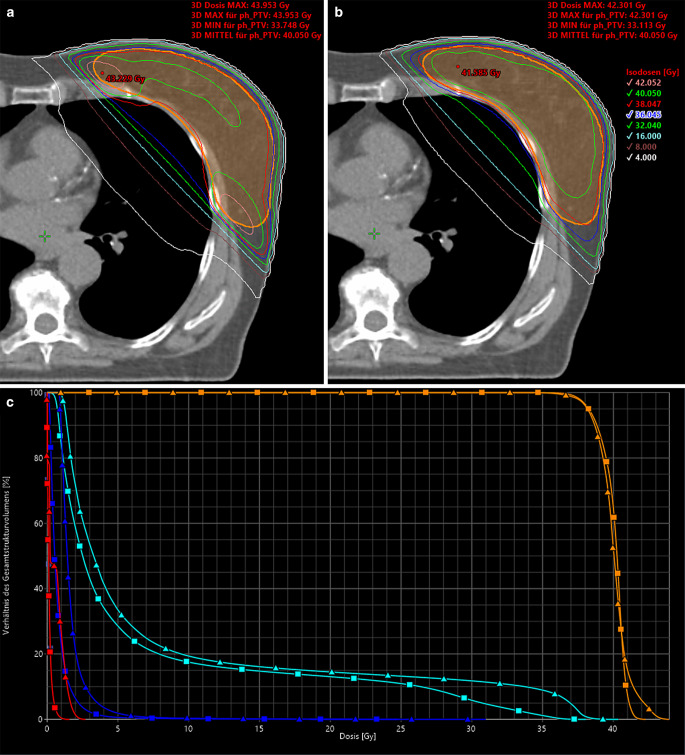
**a** Axial dose distribution of a conventional three-dimensional radiotherapy (3D-CRT) treatment plan. Tangential opposing fields with mixed beam energies (6 MV and 15 MV) and hard wedge filters were used. **b** Axial dose distribution of a hybrid volumetric modulated arc therapy (H-VMAT). Tangential opposing fields (6 MV) without hard wedge filters were used as base plan for VMAT optimization. VMAT photon energy was set to 6 MV, which corresponds to the H‑VMAT combination HV2 (Table 1). **c** Dose–volume histogram: 3D-CRT (■) versus H‑VMAT treatment plan (▲). Color selection: *orange:* PTV; *light blue:* ipsilateral lung; *dark blue:* heart; *red*: contralateral breast

Some treatment techniques could not fulfill the dose constraints D_5_ = 144–240 cGy of the contralateral breast according to the guidelines of RTOG Report 1005 (Fig. 3, Table 3). All considered hybrid combinations showed advantages over the 3D-CRT treatment plan (left D_5_ = 200 ± 33 cGy, right D_5_ = 233 ± 45 cGy). The lowest dose values with the greatest advantages were achieved with H‑VMAT combinations without the inclusion of HWF. HV2 (left D_5_ = 70 ± 23 cGy, right D_5_ = 96 ± 35 cGy) and HV3 (left D_5_ = 84 ± 29 cGy, right D_5_ = 111 ± 40 cGy) provided the best values on both breast sides.

The comparison of hybrid combinations showed a possibility to reduce the OAR doses (Table 4). Significant advantages of combinations without the use of HWF in the base plan were found for all OAR. Regarding the VMAT supplementary dose, there is a tendency that the choice of higher beam energy (15 MV instead of 6 MV) is disadvantageous for OAR exposure. For IL V4, IL D_mean_, and CB D_5_, significant advantages could be observed using a lower VMAT energy.

### Planned monitor units and beam-on times

Increased MU were required when using HWF (Table 3). Therefore, Ref and the combinations HV4 and HV5 in hybrid planning showed the highest values. Compared to Ref, the H‑VMAT plans showed consistently higher values for beam-on times. The longest BOT were observed using HWF (HV4 and HV5).

## Discussion

To our knowledge, this is the first study to analyze different base plan designs using 3D-CRT, in which the use of HWF and beam energies were varied. Variations in beam energy for the VMAT supplementary dose were also performed. The H‑VMAT combinations HV2 and HV3 (Table 1) uniformly achieved the best values regarding all dosimetric parameters in comparison to the reference treatment plan (Fig. 4; Table 3). Both favored combinations contained a 6-MV photon base plan without the use of HWF, which are conventionally used for shape compensation and dose homogenization. The comparison of different hybrid combinations showed significant disadvantages for quality indices and OAR doses when using HWF in the base plan (Table 4). In a tangential field arrangement using wedge orientation for breast surface compensation, the total body and OAR doses were increased. Advantages of virtual wedges over hard wedge filters are reported in the literature [18–21]. Less scattering effects led to lower average doses outside the irradiation field. Consequently, smaller low-dose areas in the body and lower OAR exposure, e.g., of the contralateral breast in breast radiotherapy, were observed. Furthermore, a reduction in MU was achieved. Nevertheless, virtual wedges were omitted in our study because the collimator would have to be rotated by 90 ° for technical realization (Varian TrueBeam linear accelerator). This would have complicated the exact fitting of the MLC to the PTV contour and would have increased the exposure of the OAR, especially the ipsilateral lung. The use of HWF is not necessary in H‑VMAT for reasons of dynamic supplementary dose and may even be harmful due to the increased MU and beam-on time. Furthermore, higher photon energies in VMAT showed an unfavorable tendency with regard to OAR exposure (Table 3). The maximum of depth dose curves shifts the maximum dose deeper into the tissue and a longer dose extension is created, which also increases the total body and OAR doses.

In the present study, the weighting between the radiation techniques 3D-CRT and VMAT was uniform for all combinations (80/20). Other studies confirmed the benefit of this ratio when using the H‑VMAT technique. Balaji et al. [22, 23] analyzed different ratios of chest wall radiation and identified 80/20 and 70/30 as optimal weighting concerning dose coverage of the PTV and OAR exposure. Nevertheless, the choice of weighting should be adapted individually, depending on the clinical scenario. Patient age plays an important role, as late side effects are negligible in older patients, but both acute and late effects should be considered in younger patients. With the increase of the VMAT component weighting, the low-dose area expands. In younger patients, these areas can be minimized by choosing 80/20.

The study of Lin et al. [24] showed benefits of the H‑VMAT technique over pure-VMAT and a fixed-field IMRT technique in radiotherapy of left breast cancer. They used a base plan consisting of tangential IMRT fields (T-IMRT). In our study, 3D-CRT was used in the base plan, which enables a reduction in MU and delivery time compared to dynamic techniques like IMRT. Less MLC radiation leakage and internal body scattering can be the benefit of decreased MU, which leads to a reduction in the total body dose [25]. With a reduction of the delivery time, patients have to complete fewer breathing cycles per fraction, which makes the treatment more feasible when using DIBH.

For the supplementary dose, various concepts may be used. Balaji et al. [26] reported clinically similar results of H‑IMRT and H‑VMAT using 6‑MV flattening filter-free (FFF) photon beams. The researchers recommend H‑VMAT for hypofractionated breast RT in DIBH due to reduced MU and treatment delivery time. Another study by Chen et al. [27] consistently reported advantages of H‑VMAT over H‑IMRT with regard to dose conformity, heart dose, and delivery time. They compared H‑VMAT with two different H‑IMRT designs in the treatment of early-stage left-sided breast cancer. Two tangential beams were used as a base plan and two coplanar 90-degree arcs (H-VMAT) or four IMRT fields (H-IMRT) for the supplementary dose. Ramasubramanian et al. [28] analyzed the influence of different arc designs for left breast therapy, comparing two partial arcs (2A), four partial arcs (4A), and four tangential arcs (TA). The dosimetric superiority of PTV and OAR doses could be analyzed in 2A and 4A, with the 2A method additionally allowing a reduction of MU and beam-on time. We only used flattening-filter beams and a single VMAT field over the entire tangential angular range for the supplementary dose, such that our approach resembles the 2A method. This approach may realize a further shortening of treatment time due to the uninterrupted continuous arc.

The basis for proper dose application with H‑VMAT to the patient is the existence of good reproducibility of the patient positioning. Dynamic techniques like H‑VMAT may be prone to intra- and interfractional positioning uncertainties, which demand reproducibility of patient positioning both in treatment planning and execution [27, 29–31]. In order to minimize positioning uncertainties and deviations between planned and delivered dose distributions, CT acquisition and radiotherapy should be carried out in DIBH. Besides, studies have shown benefits of DIBH concerning irradiated cardiac volume and secondary lung cancer risk in left-sided breast radiotherapy [7–10, 32]. Efficient therapy in DIBH, however, requires special measures and skills. Employee training, patient selection criteria, patient coaching, and protocols for verifying the treatment are essential requirements for efficient therapy [33].

Another key limitation of our study is the restricted patient selection. We evaluated and analyzed standardized breasts or PTV sizes only. Anatomical deviations in size or non-conventional shapes may change the findings.

## Conclusion

Our study showed the advantages of the H‑VMAT technique with a weighting of 80% 3D-CRT/20% VMAT compared to the conventional static tangential radiation technique. The combination of static and dynamic radiation techniques improved PTV dose coverage, conformity, and homogeneity. The doses of the OAR ipsilateral lung, heart, and contralateral breast were significantly reduced (*p* < 0.05) by a suitable choice of the plan combination of H‑VMAT. In treatment planning, a photon energy of 6 MV should be preferred for both the tangential radiation fields of the base plan and the VMAT supplementary dose. The use of radiation wedges in the base plan was found to be disadvantageous for PTV dose coverage and conformity, OAR doses, MU number, and delivery time.

## References

[1] Abe O, Abe R, Enomoto K (2005). Effects of radiotherapy and of differences in the extent of surgery for early breast cancer on local recurrence and 15-year survival: an overview of the randomised trials. Lancet.

[2] Darby S, McGale P, Correa C (2011). Effect of radiotherapy after breast-conserving surgery on 10-year recurrence and 15-year breast cancer death: meta-analysis of individual patient data for 10 801 women in 17 randomised trials. Lancet.

[3] Dogan N, Cuttino L, Lloyd R (2007). Optimized dose coverage of regional lymph nodes in breast cancer: the role of intensity-modulated radiotherapy. Int J Radiat Oncol Biol Phys.

[4] Kestin LL, Sharpe MB, Frazier RC (2000). Intensity modulation to improve dose uniformity with tangential breast radiotherapy: initial clinical experience. Int J Radiat Oncol Biol Phys.

[5] Hall EJ (2006). Intensity-modulated radiation therapy, protons, and the risk of second cancers. Int. J. Radiat. Oncol. Biol. Phys..

[6] Mayo CS, Urie MM, Fitzgerald TJ (2005). Hybrid IMRT plans - concurrently treating conventional and IMRT beams for improved breast irradiation and reduced planning time. Int J Radiat Oncol Biol Phys.

[7] Boda-Heggemann J, Knopf A-C, Simeonova-Chergou A (2016). Deep inspiration breath hold-based radiation therapy: a clinical review. Int J Radiat Oncol Biol Phys.

[8] Corradini S, Ballhausen H, Weingandt H (2018). Left-sided breast cancer and risks of secondary lung cancer and ischemic heart disease: effects of modern radiotherapy techniques. Strahlenther Onkol.

[9] Hepp R, Ammerpohl M, Morgenstern C (2015). Deep inspiration breath-hold (DIBH) radiotherapy in left-sided breast cancer - Bestrahlung der linken Brust in tiefer Inspiration und Atemanhaltetechnik (DIBH) bei linksseitigem Brustkrebs. Strahlenther Onkol.

[10] Stranzl H, Zurl B (2008). Postoperative irradiation of left-sided breast cancer patients and cardiac toxicity. Strahlenther Onkol.

[11] Bentzen SM, Agrawal RK, START Trialists’ Group (2008). The UK standardisation of breast radiotherapy (START) trial B of radiotherapy hypofractionation for treatment of early breast cancer: a randomised trial. Lancet.

[12] ICRU (2010). Report 83. Prescribing, recording, and reporting intensity-modulated photon-beam. J ICRU.

[13] Das IJ, Cheng CW, Healey GA (1995). Optimum field size and choice of isodose lines in electron beam treatment. Int J Radiat Oncol Biol Phys.

[14] Van Gellekom MPR, Moerland MA, Battermann JJ, Lagendijk JJW (2004). MRI-guided prostate brachytherapy with single needle method - a planning study. Radiother Oncol.

[15] van’t Riet A, Mak AC, Moerland MA, Elders LH, van der Zee W (1997). A conformation number to quantify the degree of conformality in brachytherapy and external beam irradiation: application to the prostate. Int J Radiat Oncol Biol Phys.

[16] Haverkamp U, Norkus D, Kriz J (2014). Optimization by visualization of indices. Strahlenther Onkol.

[17] Arbor A, Freedman GM, Arthur DW (2013) A phase III trial of accelerated whole breast irradiation with hypofractionation plus concurrent boost versus standard whole breast irradiation plus sequential boost for early-stage breast cancer. Radiat Ther Oncol Gr RTOG 1005. https://www.rtog.org/ClinicalTrials/ProtocolTable/StudyDetails.aspx?action=openFile&FileID=9366. Accessed 30 June 2020

[18] Akasaka H, Mukumoto N, Nakayama M et al (2017) A comparison of physical vs. nonphysical wedge modalities in radiotherapy. Radiotherapy, Cem Onal, IntechOpen. 10.5772/67057. https://www.intechopen.com/books/radiotherapy/a-comparison-of-physical-vs-nonphysical-wedge-modalities-in-radiotherapy. Accessed 30 June 2020

[19] Gopalakrishnan Z, Nair RK, Raghukumar P, Sarin B (2018). Dosimetric comparison of treatment plans using physical wedge and enhanced dynamic wedge for the planning of breast radiotherapy. J Med Phys.

[20] Weides CD, Mok EC, Chang WC (1995). Evaluating the dose to the contralateral breast when using a dynamic wedge versus a regular wedge. Med Dosim.

[21] Warlick WB, O’Rear JH, Earley L (1997). Dose to the contralateral breast: a comparison of two techniques using the enhanced dynamic wedge versus a standard wedge. Med Dosim.

[22] Balaji K, Yadav P, BalajiSubramanian S (2018). Hybrid volumetric modulated arc therapy for chest wall irradiation: for a good plan, get the right mixture. Phys Med.

[23] Balaji K, Balaji Subramanian S, Chandrasekaran AR, Ramasubramanian V (2018) Hybrid VMAT: an option to improve the plan quality for carcinoma breast. https://www.researchgate.net/publication/323337220_Hybrid_VMAT_An_option_to_improve_the_plan_quality_for_Carcinoma_Breast. Accessed 30 June 2020

[24] Lin JF, Yeh DC, Yeh HL (2015). Dosimetric comparison of hybrid volumetric-modulated arc therapy, volumetric-modulated arc therapy, and intensity-modulated radiation therapy for left-sided early breast cancer. Med Dosim.

[25] Clemente S, Cozzolino M, Chiumento C (2013). Monitor unit optimization in RapidArc plans for prostate cancer. J Appl Clin Med Phys.

[26] Balaji K, Balaji Subramanian S, Sathiya K (2020). Hybrid planning techniques for hypofractionated whole-breast irradiation using flattening filter-free beams. Strahlenther Onkol.

[27] Chen YG, Li AC, Li WY (2017). The feasibility study of a hybrid coplanar arc technique versus hybrid intensity-modulated radiotherapy in treatment of early-stage left-sided breast cancer with simultaneous-integrated boost. J Med Phys.

[28] Ramasubramanian V, Balaji K, Balaji Subramanian S (2019). Hybrid volumetric modulated arc therapy for whole breast irradiation: a dosimetric comparison of different arc designs. Radiol Med.

[29] Zhou S, Zhu X, Zhang M (2016). Estimation of internal organ motion-induced variance in radiation dose in non-gated radiotherapy. Phys Med Biol.

[30] Schwarz M, Van der Geer J, Van Herk M (2006). Impact of geometrical uncertainties on 3D CRT and IMRT dose distributions for lung cancer treatment. Int J Radiat Oncol Biol Phys.

[31] Court LE, Wagar M, Ionascu D (2008). Management of the interplay effect when using dynamic MLC sequences to treat moving targets. Med Phys.

[32] Sixel KE, Aznar MC, Ung YC (2001). Deep inspiration breath hold to reduce irradiated heart volume in breast cancer patients. Int J Radiat Oncol Biol Phys.

[33] Latty D, Stuart KE, Wang W, Ahern V (2015). Review of deep inspiration breath-hold techniques for the treatment of breast cancer. J Med Radiat Sci.

